# The Risk of Bullying and Probability of Help-Seeking Behaviors in School Children: A Bayesian Network Analysis

**DOI:** 10.3389/fpsyt.2021.640927

**Published:** 2021-05-14

**Authors:** Katarzyna Sitnik-Warchulska, Zbigniew Wajda, Bartosz Wojciechowski, Bernadetta Izydorczyk

**Affiliations:** Faculty of Management and Social Communication, Institute of Applied Psychology, Jagiellonian University in Krakow, Krakow, Poland

**Keywords:** bullying, children, help-seeking behavior, Bayesian networks, mental problems, aggresive behavior

## Abstract

An increase in aggressive behaviors in adolescents has been observed for a few years. The participation in bullying is associated with many psychosocial difficulties in adolescent development. On the other hand, the help-seeking behavior can be one of the most important protective factors that reduce the risk for this type of violence. The study was aimed at estimating the risk factors, as well as the protective factors of school bullying, by using the Bayesian networks to build a model allowing to estimate the probability of occurrence of the aggressive and help-seeking behaviors among school children. The focus was on individual risk/protective factors related to EAS temperament (emotionality, activity, and sociability) and variables related to the family context (level of cohesion, flexibility, family communication, and family life satisfaction). Bayesian methods have not been particularly mainstream in the social and medical sciences. The sample comprised 75 students (32 boys and 43 girls), aged 13–15 (M = 13.82; SD = 0.47). Assessment comprised The EAS Temperament Questionnaire, Family Adaptability & Cohesion Evaluation Scales FACES IV-SOR (Family Rating Scale), and Survey questionnaire. The Bayesian networks were applied. Depending on the values of the identified variables, very high *a posteriori* probability of bullying and help-seeking behaviors can be predicted. Four EAS subscales (Distress, Fear, Activity, Sociability) and two SOR subscales (Balanced Flexibility and Balanced Cohesion) were identified as predictors of bullying. Moreover, two SOR subscales (Family Communication and Life Family Satisfaction) and one EAS subscale (Sociability) were identified as predictors of help-seeking behaviors. The constructed network made it possible to show the influence of variables related to temperament and variables related to the family environment on the probability of bullying or the probability of seeking help and support. The Bayesian network model used in this study may be used in clinical practice.

## Introduction

Bullying is one of the most common phenomena related to aggression in school. In the school environment, bullying can refer to both harassment, intimidation, multiple use of one's predominance, verbal, physical, and social violence, as well as violence using modern technologies, known as cyberbullying (1–3). According to the review of Juvonen and Graham (4), ~20–25% of young people are directly involved with bullying, either as the perpetrator, the victim, or both. The meta-analysis by Estévez et al. (1) clearly indicates that bullying is a rather complex phenomenon. These behaviors are not only repetitive over time, but they also change forms in the course of development, especially during childhood and adolescence. Being a victim of bullying is an important risk factor for being the perpetrator of various forms of bullying, including cyberbullying in the future (1, 5).

Research conducted as a part of the WHO collaborative cross-national study Health Behavior in School-Aged Children (HBSC) on the group of 11-, 13-, and 15-year olds shows that in most countries, boys are more likely to use violence in the form of bullying. On the other hand, the proportion of boys and girls who are victims of bullying is approximate. However, according to the indicated report, girls are more often victims of cyberbullying (6). The most interesting reports show that bullying is most intense in the group of adolescents between 12 and 15 years of age, and it tends to decrease with subsequent years (2). The international HBSC report points to gender differences in this scope. Most often, 15-year-old boys and 13-year-old girls in various European countries admit to using bullying and school violence. In turn, the victims of bullying are mainly younger children and adolescents (6). However, the prevalence of these behaviors varies depending on the country and region, although, there are very few research reports in this scope.

Several theories about bullying have arisen; each one emphasizes a selected individual or environmental characteristic as the most important explanatory factor. Genetic theories indicate, based on twin studies, that the genetic factor explains more than 70% of the variance in being a victim of violence and over 60% of the variance in being a violent perpetrator (7). The Developmental Psychopathology Theory points to insecure attachment that plays an important role in shaping interpersonal relationships, and research shows that it plays a role in perpetrator of bullying in particular (8). On the other hand, the Group Socialization Theory points to the within-group process and between-group process dynamics of interactions generating bullying phenomena (e.g., group norms, standard intra-group dynamics, identification with one's own group, and competition and struggle with other groups) (9, 10). Systemic theories have pointed out that bullying and aggression among children and adolescents are multidimensional phenomena in which the importance of circular (bi-directional) intra-family interactions, as well as family messages regarding aggression, authoritarian or permissive parenting styles, disturbed communication patterns, high level of conflicts, or lack of emotional involvement in the family system were emphasized (11). However, neither of these theories has been sufficient to explain the bullying phenomenon.

### Risk Factors and Protective Factors of Bullying

Previous studies on bullying, allowed to characterize the most important risk factors for all three “actors” of bullying: perpetrators, victims, and those who are both in these two roles. Among them, there are mainly individual and family factors.

Low level of empathy, moral distancing, low awareness of the threat of media messages, in the Internet, problems with emotional regulation, or low level of family support are factors indicated as the main predictors of bullying agency (12–14). However, very interesting is the fact that among the individual risk factors of this type of behavior, the importance of low self-esteem, low level of social skills, with deficiencies in social information processing, and low sociometric status in the peer group is indicated (15, 16). Such factors also turn out to be important in the development of experiences as victims of bullying. They confirm the report by Ref. (17). They indicated a positive correlation of emotional, behavioral, and partly social difficulties both with the perpetration of violence in the form of bullying and with being a victim of this type of violence.

Noteworthy is the longitudinal study by Natesan et al. (16), carried out on a large sample (*n* = 11,715), which collected data from children, teachers, and parents. The results obtained by authors indicated that internalizing behaviors are a predictor of being a victim of school violence, while externalizing behaviors and passive communication between parents (mutual criticism, inhibition of discussion, not talking to each other) are a predictor of being a school aggressor. The researchers also noted that externalizing behaviors are more easily noticed by the environment. This seems important in the context of possible prevention of school violence victims. The authors presume that externalizing behaviors concentrate attention, and therefore are easy to see, while internalizing behaviors of nature itself are “hidden”—so it is important to look for an effective way to detect them.

When trying to understand what underlies this diversified picture of the functioning of people who play the role of a victim and/or the perpetrator of bullying, the few research attempts to trace the relationship between temperamental conditions, therefore more basic ones, and the experience of violence in the course of development seem particularly promising. The reports of Farrell and Vaillancourt (18), based on studies of longitudinal adolescents, indicate that problems with emotional regulation during childhood (reduced level of self-control) are a predictor of bullying perpetration in the period of middle adolescence, which in the future may result in the aggression in the partner relationship. Difficulties in effective coping and emotional control as well as high emotional sensitivity (including a tendency to anxiety and aggressiveness) are also features indicated as factors accompanying people experiencing bullying as a victim (19). The temperament trait also seems to be an extremely important mediator in the process of benefiting from preventive and intervention measures in the case of bullying. The studies of Nocentini et al. (20) indicate that the greatest benefits of this type of interactions aimed at counteracting the spasm of bullying are achieved by school students declaring a high level of effortful control and low or medium negative emotionality. On the other hand, in the case of victimization (being a victim of bullying), positive emotionality may be an important factor contributing to gain benefits from the proposed impacts of anti-bullying intervention.

Bullying in childhood and adolescence is a difficult phenomenon to detect. It is also difficult to undergo therapeutic interventions. This is probably because both the perpetrators and the victims very often do not tell anyone about this phenomenon. A Scandinavian study (21) showed that only about 55% of students say that they have been a victim of violence against someone, and they are not always adults. They tell it to someone mostly at home (34%), to teachers (20.6%), or other adults at school (12.7%). This is worrying that taking on the role of both the perpetrator and the victim of bullying is associated with the occurrence of psychosomatic symptoms, sleep problems, depression, and other health problems, also in the future (22–25). These reports indicate the complexity of bullying and the need to consider it in a holistic manner, assuming smooth boundaries between being the victim and the perpetrator of bullying at school. It also seems that this is not a one-off reaction, but rather a specific syndrome containing a specific way of perceiving, thinking, and acting focused on situations and actions related to crossing borders.

### Help-Seeking Behaviors

The interventions reducing the severity of bullying are a protective factor against this type of symptoms (26). At the same time, it turns out that seeking help, mainly from family members and teachers, is one of the most effective coping strategies (27–29). Barker (30) emphasizes that help-seeking behavior related to personal stress or problems is a specific, psychosocial need of young people. However, there is no general rule. Yablon (31) points out that many students are reluctant to ask for help when they experience bullying, which makes their difficulties overlooked for years. Interestingly, research by Shaw et al. (32) indicates that revealing bullying experiences to teachers does not necessarily have a direct impact on minimizing this type of experience, but it contributes to the reduction of internalizing problems. The relationship seems to be of key importance in this type of intervention. The study of Haataja et al. (33) showed that only one in four students who were chronically victimized turned to school staff for help. Other studies (29, 34) indicate that children who experience violence, including bullying, are relatively unlikely to tell teachers about the problem. They prefer family members and friends (29, 34).

Moreover, reports show that girls more often seek help than boys (29, 34–36). Girls probably perceive “telling” and social support to be a more effective strategy (34). Boys are more likely to blame themselves or respond with aggression to bullying (35, 36). Overall, it also appears that younger students turn to adults for help in dealing with bullying more than older students (34). This tendency might be related to different developmental needs. In addition, Smith and Shu (29) indicate in their study that about 30% of bullying victims had told no one of their problems (“culture of silence”). However, for those who had told, the outcome was seen as positive. This result corresponds with findings of Hunter et al. (34). Pupils who see the positive perspective (e.g., bullying stopping) are more likely to seek help than those who do not. Considering children's emotions and taking their concerns seriously by adults may increase help-seeking behaviors among students (34). Interesting analyses carried out on a group of Israeli high school students, however, have shown that if young people can benefit from the help of a school counselor, they are much more likely to do it, when he is also a teacher-counselor role. The authors of the translator's research mean greater accessibility and thus an invitation to a more positive relationship between the student and the support person (37). Telling about problems seems to be crucial for effective intervention and improving the situation of victims and bullies (29, 38).

The above findings are confirmed by the latest research conducted on a large sample of students in Finland (21). The results of the longitudinal structural equation model (SEM) showed that likelihood of telling an adult about bullying experience was related to female gender, lower grade level, the chronicity of victimization, perceived negative teacher attitude toward bullying (teacher not tolerating bullying), and perceived peer support for victims (classmates' tendency to defend students who are victimized). As Espelage and Swearer (39) indicate that even 80% of students need the primary prevention strategies, based on whole-school approach.

Therefore, it can be assumed that identifying risk factors and protective factors is essential for the effective prevention and therapy of children and adolescents engaging in bullying in all three roles: bullying perpetrator, bullying victim, and both: perpetrator and a victim of bullying. However, there is a lack of studies that capture the characteristics of this phenomenon in such a comprehensive manner and, at the same time, allow the research results to be translated into school practice.

### The Bayesian Network

Bayesian networks are statistical methods, guided by a slightly different way of thinking than traditional, and in psychology, they are something relatively new—although, the idea itself has been known for a long time. Testing the Bayesian hypothesis leads to a redistribution of probability between competing probability accounts. It is a graphical diagram that allows to visualize and model the relationships between different hypotheses and variables.

The essential characteristic of Bayesian methods is the use of probability for quantifying uncertainty in inferences based on statistical data analysis. Moreover, they can be used for the classification and prediction of states and events even when the data are partial or uncertain, regardless of variables' type and scale of measurement. The Bayesian network enables to visualize causal relationships between different hypotheses and pieces of information (results of a study). With the Bayesian networks, it is possible to express relations between variables in a clear way and to verify whether or not there is a causal relation from the data, without a controlled experiment (40). An event that occurred can be used to predict the likelihood that any one of several possible known causes was the contributing factor (i.e., to represent the probabilistic relationships between symptoms and disease). Bayesian networks can help in determining the effects of many variables on an outcome outperforming statistically classical linear models such as regression (particularly in determining variables' effects).

Regression assumes that all variables can take an infinite number of values, when one variable changes, all other variables remain the same, that relations between variables can be described by a function and the model is based on pre-assumptions. In Bayesian networks, all variables are included, and connections between the variables are based on how they most closely align across their probability distributions. The relations between the included variables may be complex and diverse, and the network is learned from the data; when estimating one variable's effect, all the other variables are included, and it is possible to see the influence of a piece of the information on a complex system.

In hierarchical models, such as Bayesian networks, it is possible to model simultaneously variability from the processes of interest, as well as from individuals and from items (41, 42). When Bayesian networks are used in conjunction with statistical techniques, the graphical model gives advantages for data modeling. The model encodes dependencies among significant variables in a clear way. Bayesian network can be also used to learn causal relationships and, hence, to gain understanding about a problem domain. Since the model has both a causal and probabilistic semantics, it is an ideal representation for combining prior knowledge and data (43).

In detail, a Bayesian network is a graphical model (namely, the directed acyclic graph) together with the corresponding probability potentials (43). Bayesian network is a way of structuring a situation for reasoning under uncertainty; the structure of the directed graph can mimic the causal structure of the modeled domain. A graph is constructed to represent causal relations between events (44). A set of variables and a set of directed links (also called arcs) between variables are used to form a causal network. A model can be used to show and predict how a change in one variable may change the other variables' values. A network consists of the following: a set of variables and a set of directed edges between variables; each variable has a finite set of mutually exclusive states; the variables together with the directed edges form an acyclic directed graph; a directed graph is acyclic; a conditional probability table is attached to each variable (44).

Bayesian networks allow performing Bayesian inference, such computing the impact of observing values of a subset of the model variables on the probability distribution over the remaining variables. They represent joint probability models among a given set of variables. Each variable is represented by a node in a graph. The direct dependencies between the variables are shown by directed edges between the corresponding nodes and the conditional probabilities for each variable (that is, the probabilities conditioned on the various possible combinations of values for the immediate predecessors in the network) are stored in potentials (or tables) attached to the dependent nodes (43).

In the graph structure of the probabilistic domain is included not so much information about its numerical properties. These are encoded in conditional probability distribution matrices (equivalent to the factors in the factorized form), called conditional probability tables that are associated with the nodes (40, 45). The basis for the conditional probabilities can be ranging from well-founded theory over frequencies in a database to subjective estimates (44).

The directed acyclic graph may be interpreted as follows: a directed edge between two variables shows the modeling assumption that there is a direct causal connection between the two variables, the cause-to-effect relationship indicated by the direction of the arrow. When there are some arrows, it is indicated that there is no direct causal relation between the variables. The Bayesian network may have a causal interpretation, and the dependence structure between different variables in the network is described by the structure of the directed acyclic graph (40, 44). Information about the observed value of a variable is propagated through the network to update the probability distributions over other variables that are not observed directly.

There are two basic problems, connecting with learning, in Bayesian networks, that is finding the structure of the Bayesian network from the data and, when the structure is built, learning the conditional probability potentials (40). There are several ways to address these problems and approaches to learning the structure of the network and its parameters (42, 45, 46). One of the simplest methods for general inference in Bayesian networks is based on the principle of variable elimination. It is a process in which variables from a Bayesian network are successively removed while maintaining its ability to answer queries of interest (45). Variables are eliminated if new distribution is as good as the original, which included all variables. This procedure will always work, but it is exponential in complexity in the number of variables in the Bayesian network (45), and even when unnecessary variables are eliminated, it is still unknown, what the best possible structure of the Bayesian network is.

One of the most commonly used tools to find the optimal Bayesian network is the Chow–Liu algorithm (40, 44, 46). The algorithm uses the maximum likelihood estimators of mutual information rather than the true mutual information values. Weights of each possible edge are computed, tree spanning maximum weight and directions to the edges in the maximum weight spanning tree are found [for more details, see (46)].

### The Purpose and Model of our Research

The entire study was focused on determining the possibility of using Bayesian networks to predict the behavior of adolescents related to bullying as well as seeking help in a situation of violence. The purpose of this article is to show that it is possible to meet the requirement for a structured method of building Bayesian networks (BN) to model risk of bullying and probability of searching for help behaviors among school children. Especially interesting was using raw, unaggregated data and exploring the possibility to use Bayesian networks to develop a model allowing for prediction of bullying behaviors.

In particular, the research was aimed at estimating the risk factors, as well as the protective factors of school bullying, by building a model allowing to estimate the probability of the behavior occurrence related to the use of school violence and seeking help in the situation of experiencing bullying among school-age children in clinical practice. The authors treated the phenomenon of bullying in a comprehensive manner, but also firmly rooted in the respondents' perceptions. Therefore, as a criterion for bullying, similar to the HBSC research (6), they adopted declarations about the occurrence—at least twice in the last few months—behavior of intimidating, damaging, or threatening. In turn, the criterion of seeking help from others was declarations about the presence of sources of support in the environment (family members, teachers, and peers).

As a consequence, the presented study was primarily focused on identifying predictors of school bullying by selecting tools and variables that allow for the differentiation of children involved in bullying from those who do not. The focus was on individual variables related to temperament (such as emotionality, activity, and sociability) and variables related to the family context (such as the level of cohesion, flexibility, family communication, and family life satisfaction). The aim of the research was also to determine the qualitative differences between the group of pupils resorting to bullying and those seeking help—thus, behaviors were perceived as a protective factor in the situation of bullying.

Research goals can be presented in the form of the following research questions:

- Do temperamental individual variables predict student bullying?
- Do the variables related to the family context predict student bullying?
- Do temperamental individual variables allow to predict asking for adults' help, which is considered as a protective factor for student bullying?
- Are the variables, related to the students' family environment allow for the prediction of reaching for adults help, considered as a protective factor in terms of student bullying?
- What is the likelihood of bullying depending on the severity of variables related to temperament and family context?
- What is the likelihood of protective behavior occurrence, related to seeking help in adults, depending on the severity of variables, related to temperament and family context?

## Materials and Methods

### Participants and Procedure

The study group consisted of students, attending public schools in the Silesia Region, who were invited to participate in psychological workshops on counteracting school violence. The workshops were of the nature of primary prevention. The goal was to reduce the number of new cases of bullying by developing school's positive climate and whole-school approach. The students and their guardians were contacted through their schools and were informed about the possibility of participating in workshops and the study. The participants were volunteers. The workshops consisted of five sessions and were based on open discussion and role playing. The sessions were focused on occurrence of bullying among students, including understanding the phenomenon of violence and human rights; increasing cooperation between students and group cohesion; developing a sense of responsibility; learning coping strategies; and conflict solving.

The following criteria of inclusion in the sample of respondents were used, which were verified by means of questions in the questionnaire referring directly to the following indicators:

- School age (12–15 years old), Polish nationality, attendance: primary school, junior high school, or high school (the age criterion of respondents was related to the period of high risk of aggravation of behaviors related to bullying).
- Lack of diagnosis and being subject to therapeutic interventions due to the use of violence, being a victim of violence, disclosed mental diseases and disorders, eating disorders, behavioral disorders, emotional disorders, disabilities, or neurodevelopmental difficulties.

The research was conducted in 2019. Prior to the research, consent was obtained from the legal guardian of the child/parent, as well as the teenager. It was reported that participation in the study was voluntary and anonymous. Eighty students were examined in the study, and 75 people were included in the final analyses (five questionnaires were incorrectly completed). Among the 75 examined students, there were 32 boys and 43 girls, aged 13–15 (M = 13.82; SD = 0.47). The detailed characteristics of the study group are presented in Table 1.

**Table 1:** Selected characteristics of the study group (*n* = 75).

		**Girls (*n* = 43)**	**Boys (*n* = 32)**	**Together (*n* = 75)**
Age	13 years old	9 (21%)	8 (25%)	17 (23%)
	14 years old	34 (79%)	24 (75%)	58 (77%)
Number of siblings	0 (an only child)	4 (9%)	3 (9%)	7 (9%)
	1	19 (44%)	16 (50%)	35 (47%)
	2	16 (37%)	8 (25%)	24 (32%)
	3 and more	4 (9%)	5 (16%)	9 (12%)
Parents	Together	38 (88%)	22 (69%)	60 (80%)
	Divorced	5 (12%)	10 (31%)	15 (20%)
Financial situation	Bad	0 (0%)	1 (3%)	1 (1%)
	Average	7 (16%)	7 (22%)	14 (19%)
	Good	27 (63%)	13 (41%)	40 (53%)
	Very good	9 (21%)	11 (34%)	20 (27%)

### Compliance With Ethical Standards

Ethical approval was obtained from the relevant institutional ethical review committees, and the research was conducted in accordance with national and international regulations and guidelines. All subjects gave their informed consent for inclusion before they participated in the study. The study was conducted in accordance with the Helsinki Declaration, and the protocol was approved by the Ethics Committee of Institute of Applied Psychology, Jagiellonian University in Krakow. This project is public at Open Science Framework (https://osf.io/7d3sk/?view_only=472d1e1895bc4eefbfa7341a9396b61c).

### Methods

#### The EAS Temperament Questionnaire

The EAS Buss and Plomin's Temperament Questionnaire, in the Polish adaptation of Oniszczenko (47), was used to measure the variables related to temperament. As the author recommended, for the purpose of the research, the adult version was used as an experimental version for adolescents (~14 years old) (47). The questionnaire contains 20 items for the diagnosis of temperament, which is understood as a set of inherited personality traits that are revealed early in the life of the individual. They have the character of statements, the truthfulness of which is assessed by the respondent on a five-point scale (from *definitely not* to *definitely yes*). They allow to describe the temperament on three scales:

- *Emotionality* (temperament component characterizing emotions in terms of dissatisfaction Distress–undifferentiated emotionality, a tendency to react with strong anxiety. Fear–a tendency to avoid aversive stimulation and fleeing from threat and anger. Anger–a tendency to react with anger, which is caused by stimuli that irritate or frustrate).
- *Activity* [temperament component related to the expenditure of physical energy. The definition of this feature excludes any mental effort accompanying cognitive processes and agitation related to emotional processes. The range of variability of this feature is significant, from immobility to extremely energetic behavior. The main components of activity are pace (speed of action) and vigor (related to the strength or intensity of the reaction)].
- *Sociability* (a temperamental component defined as a general tendency to seek and be with other people and avoid loneliness).

#### Family Adaptability and Cohesion Evaluation Scales FACES IV-SOR

Olson's Family Adaptability and Cohesion Evaluation Scales (48) was used to measure family-related features in the Polish adaptation of Margasiński-SOR (English: Family Rating Scales) (49, 50). The questionnaire consists of 62 statements, to which the respondent responds on a five-point scale (from *completely disagree* to *completely agree*). These theorems are grouped into eight scales. Six of them are the main scales of the Circle Model created by David H. Olson, concerning two dimensions of family functioning:

- *Coherence* (understood as an emotional bond between family members; indicators of family cohesion are: mutual emotional closeness, the quality of psychological boundaries between family members, the existence of coalitions, the amount of time spent together, common interests, and forms of rest, the size of a common circle of friends, the degree of consultation with each other on various decisions);
- *Flexibility* (defined by the quality and degree of changes taking place in the family; indicators of flexibility are the scope of taking over leadership, negotiation styles, roles adopted by family members, and rules defining relationships between family members).

The scales that arise from the values obtained in both dimensions described are balanced scales: *Balanced Cohesion, Balanced Flexibility*, and unbalanced scales, i.e., (*Disengaged, Enmeshed, Rigid, Chaotic*). The other two scales measure *Communication* (which is the third dimension of the Circle Model) and *Family Life Satisfaction*. *Family Communication* is understood as the communication skills used by a given family system. On the other hand, *The Life Family Satisfaction Scale* determines the degree to which individual family members feel happy and fulfilled with each other. It is worth noting that the *Family Communication* and *Life Family Satisfaction Scales*, as well as *Balanced Cohesion* and *Balanced Flexibility*, are characterized by the highest reliability. For this reason, and taking into account the requirements of using Bayesian networks, it was decided that the results from these four scales be used in the presented analyses.

#### Survey Questionnaire

The Survey Questionnaire was developed by one of the authors of the presented research for the purpose of the research process. It included questions about demographic data, family structure, as well as phenomena related to school violence and seeking help from other adults.

The bullying section included questions about the presence in the past few months of such behaviors as bullying, blackmailing, making fun of others, gossiping about others, theft, extorting money/things, verbal abuse, destroying school equipment, threatening someone, isolating someone in the group, hitting others, name-calling, insulting, and destroying other people's things.

The part on help-seeking behavior included questions about the perception of parents, siblings, peers, teachers, and other specialists working in the school as real and potential sources of support in the situation of experiencing bullying.

### Data Analyses

The collected data have been codified and statistically processed using Bayesian networks. When reasoning about the possibility of aggressive behaviors among school children, it would be use intuitive procedure for reasoning. It was assumed that a student can demonstrate bullying (H). The next step would be to update prior belief about H that once was observed as a child's hostile behavior (E). Updating takes into account the likelihood of the evidence, that is, the chance of observing the action E assuming that bullying (H) is true. Such process of reasoning is a perfect match for Bayesian inference (51). It was started with a prior probability P(H) for the hypothesis H. It is the conditional probability of E given H, which was written as P(E|H). To each variable, a conditional probability table P(H|E1, E2, … En) is attached (44). Whenever a statement about the probability P(H) of an event H is given, then it is implicitly given conditioned on other known factors. With the use of Bayes theorem, the prior belief about H in the light of observing E was updated. In other words, Bayes calculates P (H|E) in terms of P(H) and P(E|H). Bayes' rule ensures a method for updating beliefs about an event (H) given, taking into consideration that it is given an information about another event (E). For this reason, P(H) is usually called the prior probability of A, whereas P(H|E) is called the posterior probability of H given E; the probability P(H|E) is called the likelihood of H given E (44).

There were only 75 students examined in the study. In general, the quality of the Bayesian network's estimates improves as the sample size increases; the absolute error is bounded as the sample size tends to infinity. On the other hand, it is argued (44) that reliable and limited predictions of network architecture constructed under constrained sample sizes have the potential to generate more efficient network. In exploratory studies, the Bayesian network does not computationally scale well to large numbers of features—larger samples usually lead to more complex networks. We have assumed that the study of 75 participants will allow us to construct an efficient and simple Bayesian network useful in everyday practice.

## Results

Collected data shows that 65.3% of students participating in the study were laughing at others, 60% of the study participants were calling names, 29.3% were using isolation, 24% were beating, 20% confessed to destructions of equipment, 16% of participants used sexual insults, 9.3% used blackmailing, 8% used threats, in 5.3% case phishing was reported, and 1.3% of the study group committed theft.

Most of the participants seeking help from others chose their mother (54.7%), only around one third searched from support from the father (29.3%), and around 25% searched from support of one of the siblings. Of the study participants, 48% found support in peers, 29.3% received help from a tutor, 26.67% from school pedagogue, 13.3% from the school's principal, 9.3% from teachers, and 4% from the religion teacher.

### Bullying Predictors

In the surveyed group, 18 students declared the presence of harmful behaviors related to bullying toward others. To explore the differences between the groups, mean comparison tests were carried out. Since there were significant differences between the sizes of the groups, and the distribution of the studied variables did not meet the conditions for normal distribution, the non-parametric Mann–Whitney *U*-test was used for further analysis. Descriptive statistics for research variables in the groups of the bullies and non–bullies are presented in Table 2.

**Table 2:** Descriptive statistics for groups of bullies (*n* = 18) and non-bullies group (*n* = 57).

**Variable**	**Bully (*****n*** **=** **18)**		**Non-bully (*****n*** **=** **57)**		***U***	***p***
	**M**	**SD**	**M**	**SD**		
EAS distress	11.28	3.10	9.17	2.43	317.00	0.01
EAS fear	9.89	3.41	9.67	2.79	497.00	0.85
EAS anger	11.55	3.55	11.37	2.78	468.00	0.58
EAS activity	12.33	3.10	12.33	2.38	495.50	0.83
EAS sociability	15.17	3.36	16.56	2.24	403.50	0.17
EAS emotionality	32.72	7.59	30.21	6.02	414.50	0.22
SOR A balanced cohesion	28.78	5.27	28.26	5.15	461.50	0.52
SOR B balanced flexibility	23.56	4.15	24.42	4.84	453.50	0.46
SOR G family communication	37.17	8.79	36.79	9.13	512.00	0.99
SOR H family satisfaction	38.83	9.03	37.98	7.41	456.50	0.49

*EAS, The EAS Buss and Plomin's Temperament Questionnaire; SOR, Olson's Family Adaptability and Cohesion Evaluation Scales; M, mean, SD, standard deviation*.

### Bayesian Networks and Probability of Bullying

**Figure 1:**
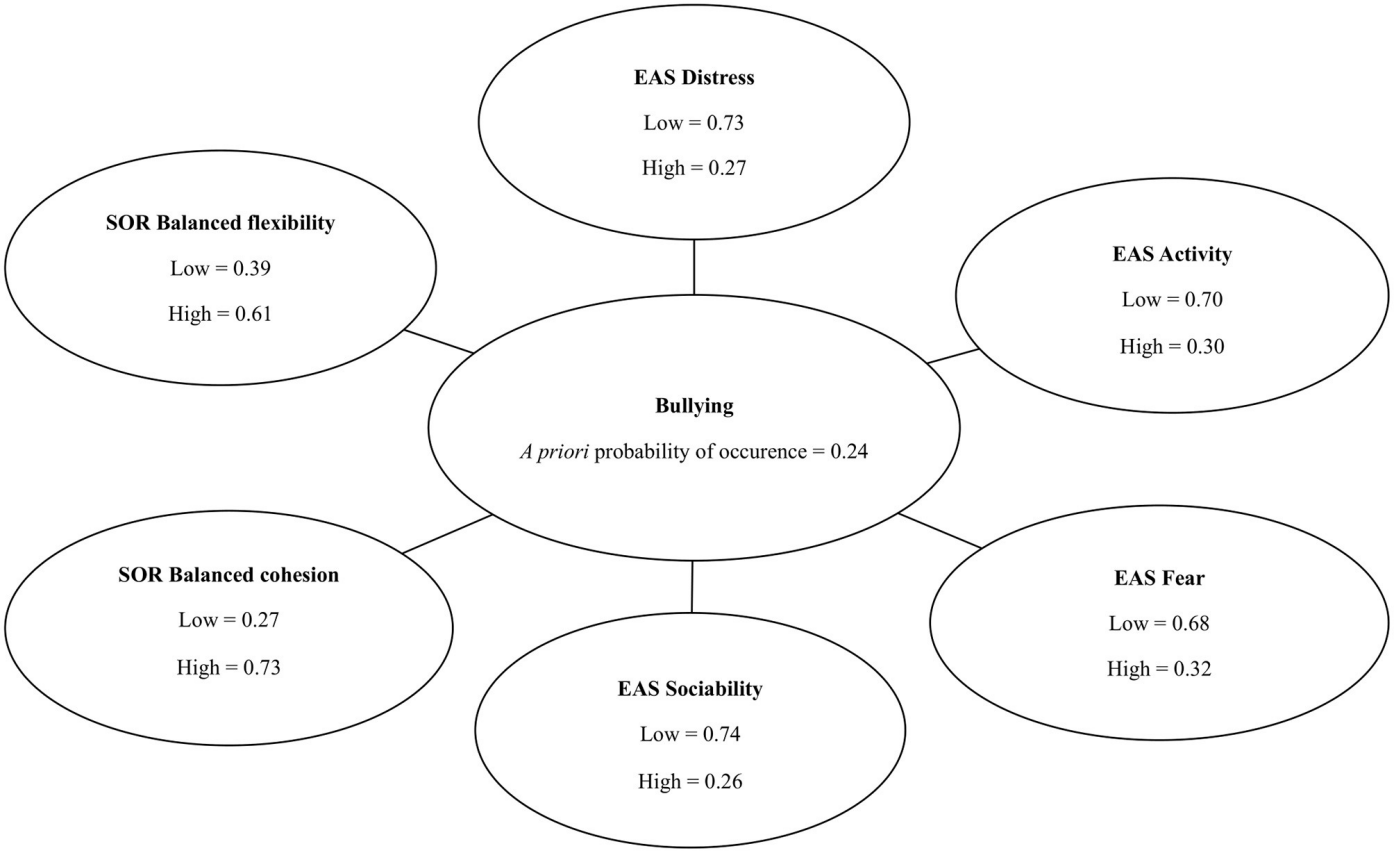
*A priori* overall probability of bullying and distribution of values of the predictor variables.

**Figure 2:**
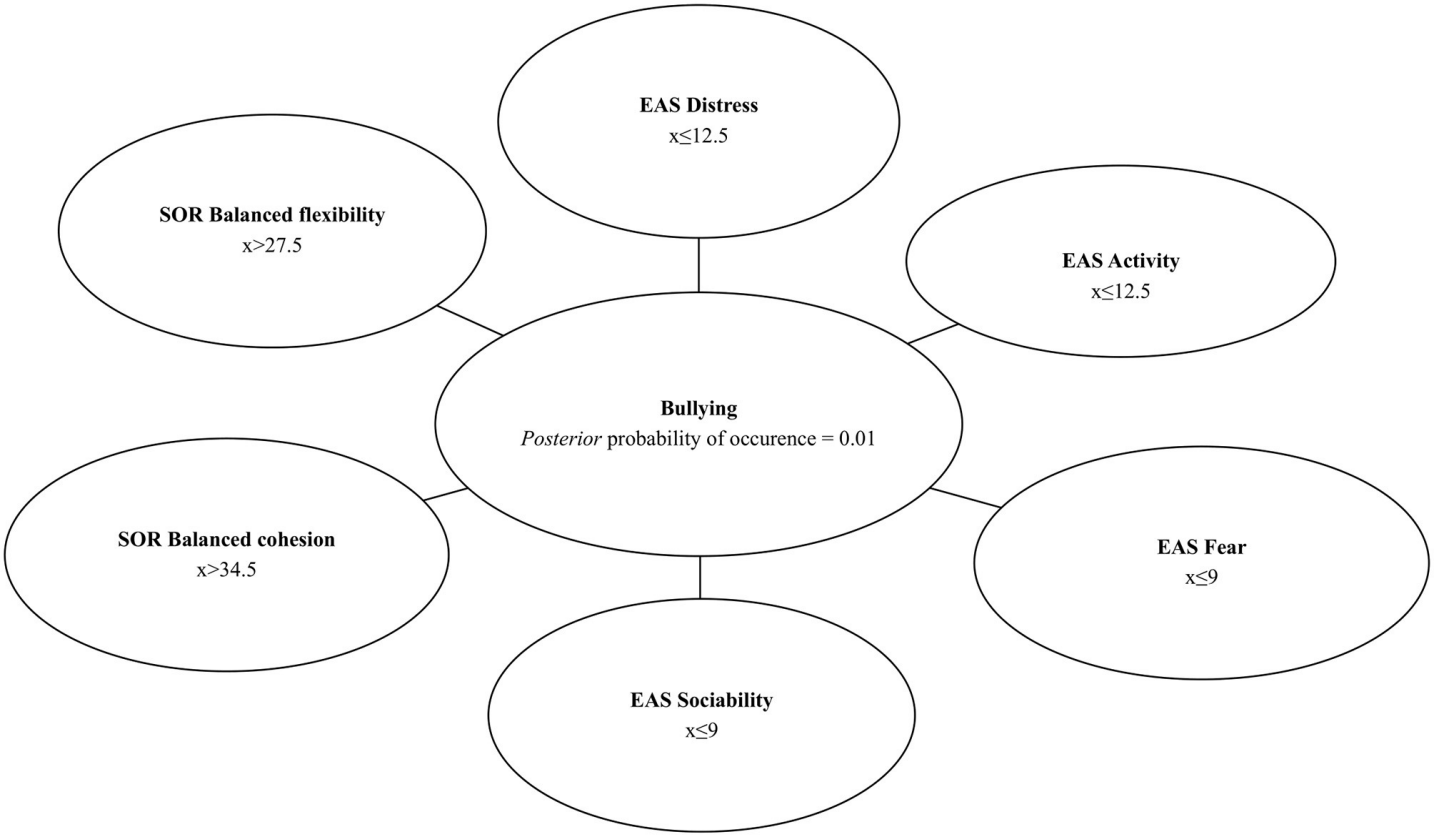
Bayesian network–low *posteriori* conditional probability of bullying.

**Figure 3:**
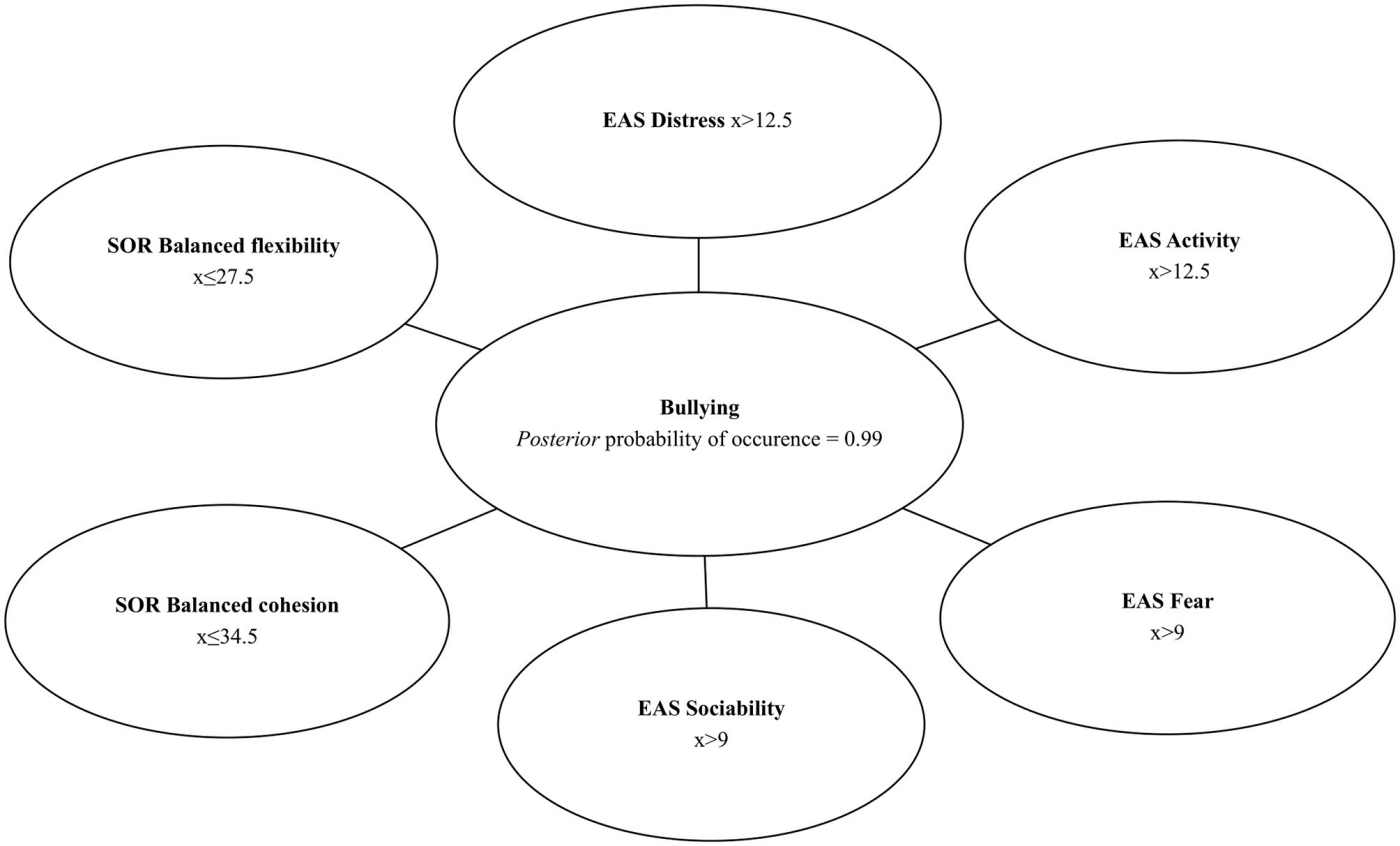
Bayesian network–high *posteriori* conditional probability of bullying.

Taking into consideration that 18 of 75 school children participated in the study admitted to behaviors harmful to others, it is possible to estimate *a priori* probability of bullying among students as P_bullying_ = 0.24 (Figure 1). The Chow–Liu algorithm (46) was used to build a Bayesian network using observed values of the selected EAS and SOR subscales to establish probability distribution of bullying. Four out of six EAS subscales (*Distress, Fear, Activity, Sociability*) and two out of four SOR subscales (*Balanced Flexibility* and *Balanced Cohesion*) were identified as predictors of bullying. Depending on the values of the identified variables, very high *a posteriori* probability of bullying (P_bullying_= 0.99) or very low *a posteriori* probability of bullying (P_bullying_ = 0.01) can be predicted. If measurement with the use of SOR and EAS reveals that a student receives the following scores: more than 12.5 in the EAS *Distres*s, more than 9 in the EAS *Fear* subscale, more than 12.5 in the EAS *Activity* subscale, and more than 9 in the EAS *Sociability* subscale, a score of less or equal to 34.5 in the SOR balanced cohesion subscale and less or equal to 27.5 in the SOR *Balanced Flexibility*—*a posteriori* conditional probability of bullying is equal to 99% (P_bullying_ = 0.99). However, when a participant receives a result ≤ 12.5 in the EAS *Distress* subscale, ≤ 9 in the EAS *Fear* subscale, ≤ 12.5 in the EAS *Activity* subscale, ≤ 9 in the EAS *Sociability* subscale, score of more than 34.5 in the SOR *Balanced Cohesion* subscale and more than 27.5 in the SOR *Balanced Flexibility*—*a posteriori* conditional probability of bullying is marginal, equal to 1% (P_bullying_ = 0.01). In case of results partially overlapping, intermediate probabilities can be expected. These results are presented in Figures 2, 3.

### Predictors of Tendency to Seek Help

The sample consisted of 75 school children, in which 72 declared that they had persons, who can be asked for help or support, and three stated they have not been seeking help. To explore the differences between the groups, mean comparison tests were carried out. There were significant differences between the sizes of the groups, and the distribution of the studied variables did not meet the conditions for normal distribution; the non-parametric Mann–Whitney *U*-test was used for further analysis. Descriptive statistics for research variables in the group of children searching for help or support and group of children not seeking for help are presented in Table 3.

**Table 3:** Descriptive statistics for groups of school children searching for help or support (*n* = 72) and children not seeking for help or support (*n* = 3).

**Variable**	**Seeking help (*****n*** **=** **72)**		**Not seeking help (*****n*** **=** **3)**		***U***	***p***
	**M**	**SD**	**M**	**SD**		
EAS distress	9.62	2.67	11.00	4.59	92.00	0.67
EAS fear	9.69	2.94	10.33	3.21	93.50	0.70
EAS anger	11.37	2.84	12.33	6.03	90.50	0.64
EAS activity	12.29	2.59	13.33	1.53	80.50	0.46
EAS sociability	16.29	2.51	14.67	4.62	77.00	0.41
EAS emotionality	30.69	6.42	33.67	8.50	82.50	0.50
SOR A balanced cohesion	28.71	4.93	20.67	4.94	22.50	0.02
SOR B balanced flexibility	24.35	4.72	21.00	2.00	59.00	0.19
SOR G family communication	37.35	8.58	25.67	13.65	38.00	0.06
SOR H family satisfaction	38.37	7.86	33.67	2.89	56.00	0.16

*EAS, The EAS Buss and Plomin's Temperament Questionnaire; SOR, Olson's Family Adaptability and Cohesion Evaluation Scales; M, mean, SD, standard deviation*.

### Bayesian Network and Probability of Searching for Help or Support

**Figure 4:**
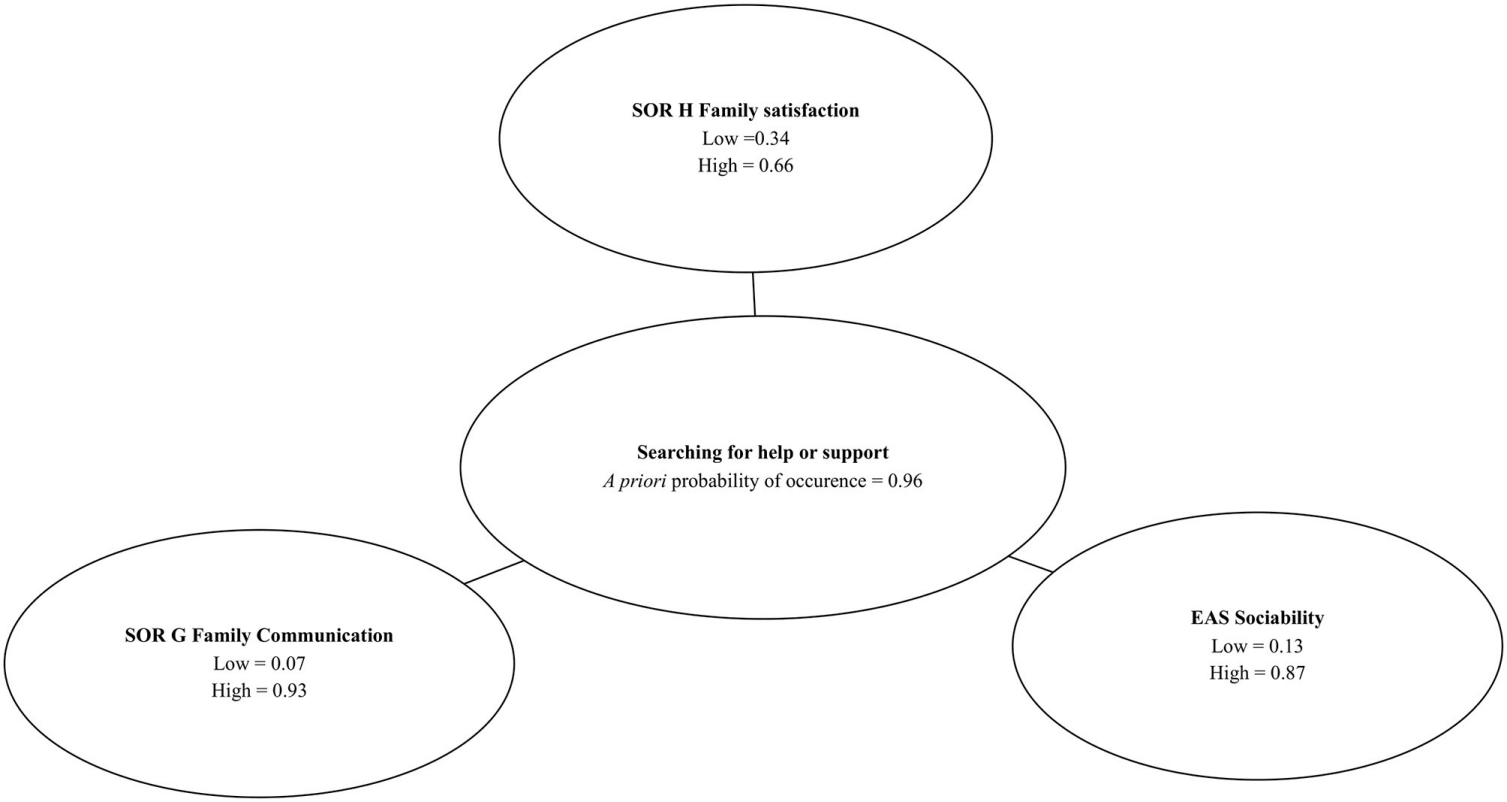
*A priori* probability of searching for help or support and distribution of values of the predictor variables.

A vast majority of participants presented help or support-seeking behaviors (96%). However, to test the applicability of the Bayesian networks, we also used the Chow–Liu algorithm to build a model describing relations between searching for help or support and psychological variables measured with SOR and EAS (Figure 4).

**Figure 5:**
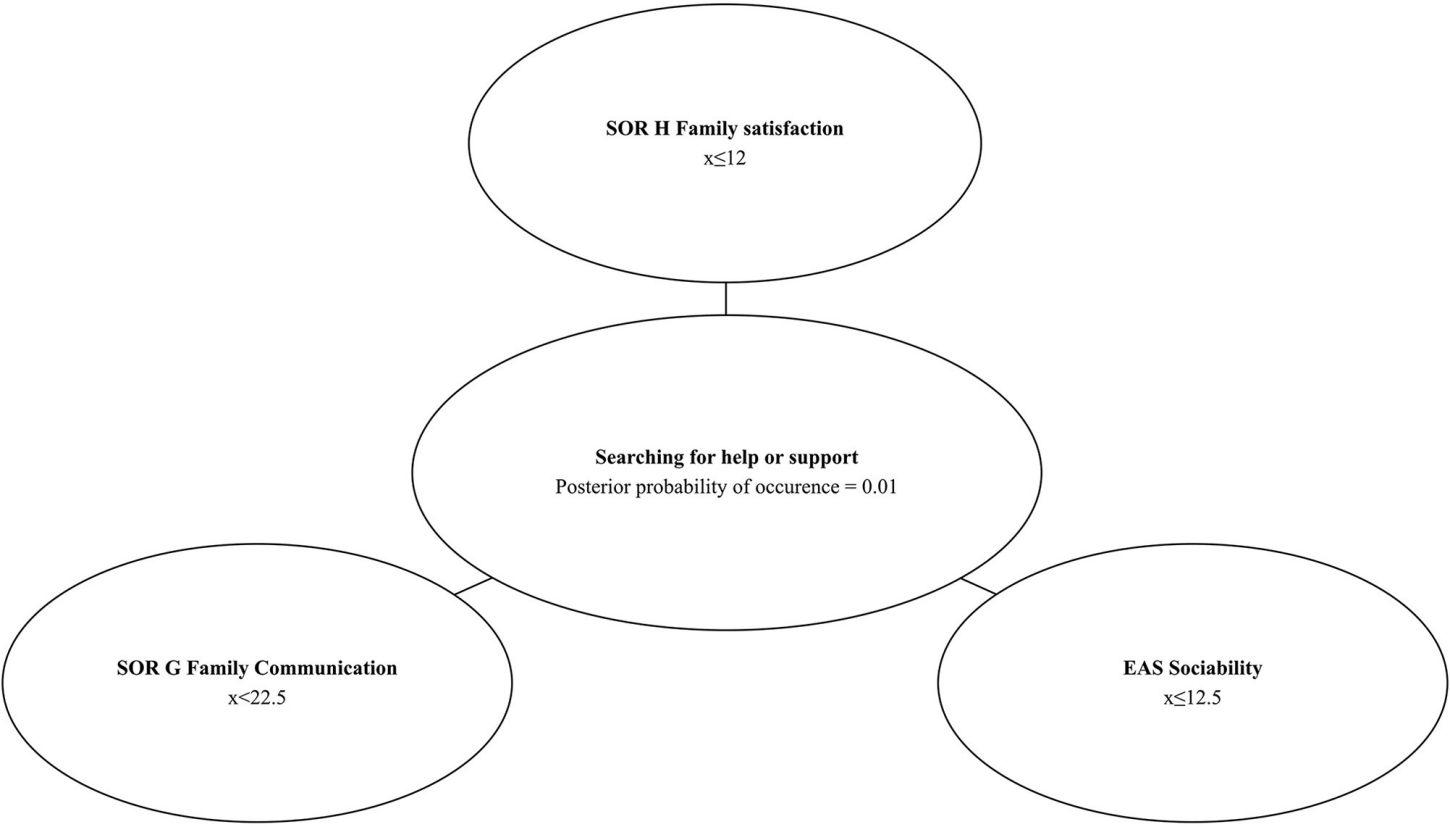
Bayesian network–low *posteriori* conditional probability of searching for help or support.

**Figure 6:**
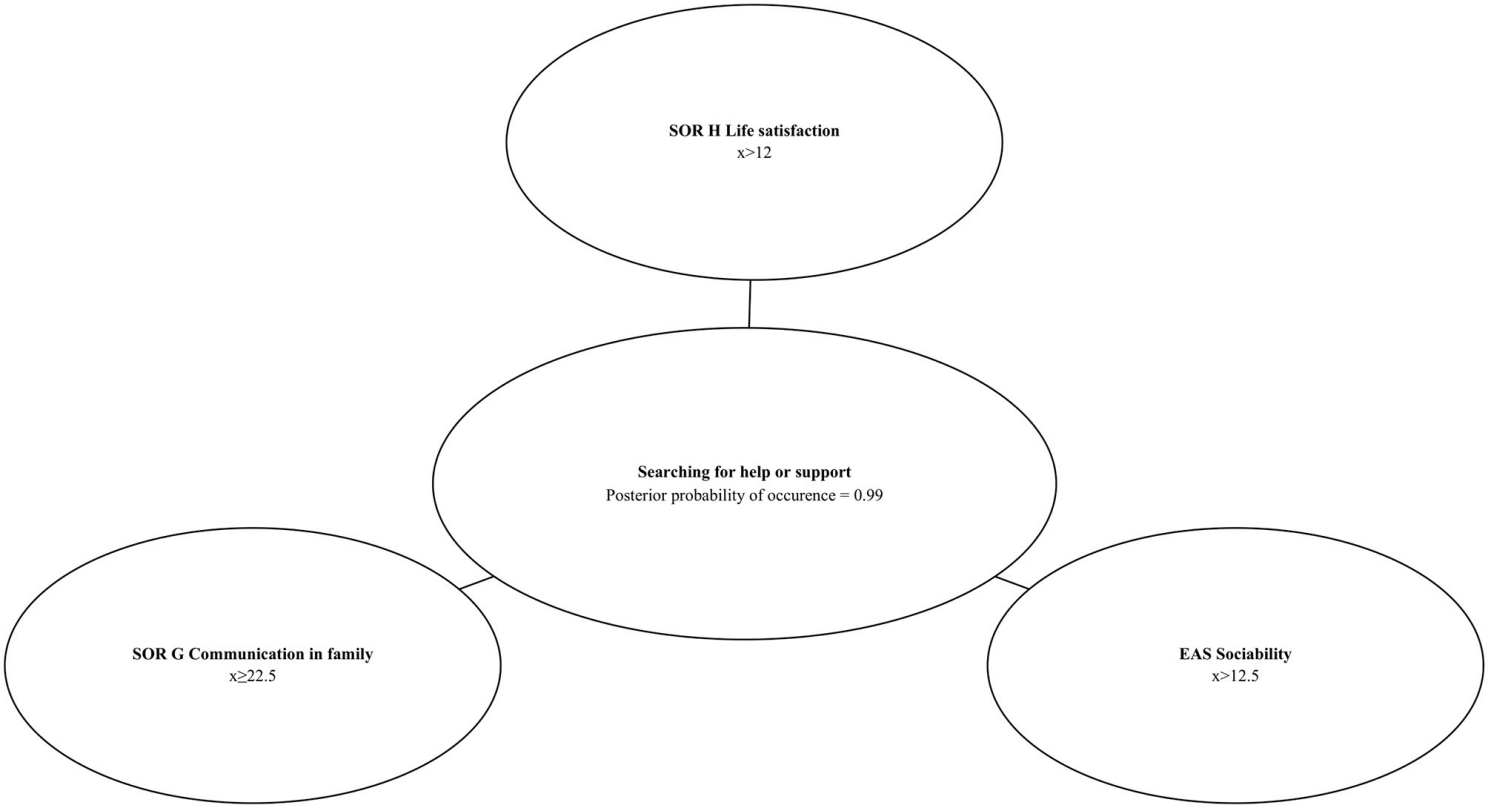
Bayesian network–high *posteriori* conditional probability of searching for help or support.

Two out of four SOR subscales (*Family Communication* and *Life Family Satisfaction*) and one EAS subscale (*Sociability*) were identified as predictors of help-seeking behaviors. The Bayesian networks allows to predict with 99% *a posteriori* probability (P_searchingforhelporsupport_ = 0.99) that a school student will search for help or support when results of the diagnosis show: SOR *Life Family Satisfaction* higher than 12, SOR *Family Communication* higher or equal to 22.5, and EAS *Sociability* subscale's value higher than 12.5. It can be expected, with 1% conditional probability (P_searchingforhelporsupport_ = 0.01) that a child will be searching for help or support from the others when the SOR *Family Life Satisfaction* subscale's value is ≤ 12, SOR *Family Communication* subscale's value is lower than 22.5, and EAS *Sociability* subscale's value is equal or lower than 12.5. These results are presented in Figures 5, 6.

## Discussion

In this article, we have outlined a general framework for modeling data, based on Bayesian networks. The paper focuses on how Bayesian networks can capture violent behavior risk assessment representing the probabilistic causal relations between psychological traits and behavior. This approach allows us to model large bodies of interrelated personal characteristics and behaviors, and capture inference patterns. We have used the Bayesian networks to provide a normative model for representing and drawing inferences from psychological traits, supporting the task of assessing risk of bullying and the probability of help-seeking behaviors among school children. The Bayesian networks representing *a posteriori* conditional probability of bullying or searching for help and support, referring to selected subscales of the EAS Temperament Questionnaire (47) and the Family Adaptability & Cohesion Evaluation Scales FACES IV-SOR (49) allow to estimate probability and accurately predict behavior. Models can be also used as a plausible explanation (representation) that explains factors determining school children' actions.

Among the factors that make it possible to predict the likelihood of bullying behavior toward others are those related to temperament and the family environment. The research conducted by the authors showed that the high intensity of bullying behavior is fostered by a high level of anxiety, avoidance, and aversion, as well as active in the context of high physical energy expenditure. These are aspects directly related to emotional regulation and reactivity, which in adolescence have a direct impact on behavior in the social environment (52). These results are also compatible with the reports of other researchers, including that of Marini et al. (53) or the already mentioned Farrell and Vaillancourt (18), indicating a weakened self-regulation ability and a high level of arousal in bullying perpetrators. Earlier studies by Bacchini et al. (54) also draw similar conclusions. They indicate that temperamental factors such as the lower inhibitory control, negative emotionality, or problems with the regulation of own emotions and behavior can be both a trigger factor and a significant risk factor for bullying activities by schoolchildren.

In such situations, bullying can be adaptive. For example, they serve to regulate the voltage, allowing you to experience power or your attractiveness [see (55, 56)]. In this context, it can be seen as a behavioral expression of temperament or as a strategy for achieving emotional and social goals. Farrell and Vaillancourt (18) also pointed to this kind of thesis. This thesis seems even more valid in the context of the authors' observations of the presented research with a high probability of bullying with a high tendency to seek social relationships and stay among people. This could confirm the thesis about the adaptive function of bullying as a basis for experiencing one's attractiveness—thus, satisfying the aspiration to associate. In connection with the indicated tendency to react with anxiety, the tendency to bullying may also be interpreted as a strategy for a neurotic drug. This assumption is partially confirmed by reports by Alonso and Romero (57), which indicate the presence of higher rates of neuroticism in adolescents who play the dual role of aggressor–victim. However, this issue requires further research.

It is worth noting that the effect of difficulties in regulating one's own emotional states may be quite permanent. This means that, therefore, the behavior of bullying presented and potentially related to this aspect may have a tendency to persist. Other ways of regulating tension or achieving social gains may then not be available. As indicated by Farell & Vaillancourt (18) in their research, this is a complex phenomenon and may be related to the entire system of co-occurring risk factors, including frustration, problems with inhibitor control, and bullying (and not the intensity of a single factor).

As presented in our research, factors related to the family environment are also factors that increase the probability of bullying in accordance with the Bayesian network analyses. A low level of sustainable flexibility and a low level of sustainable consistency favor bullying behavior. According to Olson's model, such features of family functioning are characteristic of problem families, although the type and nature of problems depend on how other features of the family system are shaped (48–50). Low scores on the *Balanced Consistency* and *Balanced Flexibility Scales* thus determine the families in the risk group; not yet unconnected and tangled, or rigid or chaotic, but with problems with emotional closeness or effective adaptation to changes, especially in situations of challenges, difficulties, and crisis (48). At the same time, there are not many studies that consider this topic and explain the relationship between this type of functioning and bullying in children and adolescents.

However, there are reports suggesting that bullies experience less emotional involvement and conflict in their parents' relationship (58). The report of Önder and Yurtal (59) indicates that the problem factors in the family environment, which is conducive to bullying behavior in young people, may be, first of all, problems with effective problem solving, impaired communication skills in the family, inconsistent relationship with parents, disproportionate or ineffective division of roles in family, or a lowered level of emotional responsiveness, a lowered level of emotional involvement, or a weakened control of behavior manifested in inconsistent or ineffective educational methods. It is worth noting that the authors' research referred to a similar perception of two students in grades 7 and 8 of primary schools involved in the bullying phenomenon: people exhibiting bullying behavior, as well as those who are victims of bullying. The already cited studies by Wolke and Lereya (23) or the earlier analyses by Bowes et al. (60) drew attention to a similar aspect. They revealed that people who were both perpetrators and victims more often than other children experienced abuse, neglect, or inadequate parental care.

This type of parenting environment also does not seem to be a source of support. In adolescence, with overlapping crises and developmental stresses, it may predispose to increasing frustration, loneliness, and seeking self-evaluation through behavior that gives advantage over others, a sense of strength and domination. Bullying is one of them. Papanikolaou et al. (61) indicated in their research that a significant correlation occurs between the lack of adequate support, mainly from the mother, and engaging in bullying behavior at school.

Experiencing support and understanding from loved ones, and also from other adults, is essential for balanced development. The availability of this type of support may increase the sense of security, especially in the period of middle adolescence, where youth are not completely independent yet, but are supposed to be not dependent any more. Support, without excessive interference or control, is extremely important for the development of self-confidence, self-esteem, and importance. The prospect of this type of support or the perception of this type of support is undoubtedly protective. The analyses of Otake et al. (62) indicate that the experience of being left behind by loved ones, even for economic and professional reasons (left-behind children), is conducive to using bullying behavior (as well as finding oneself in the role of a bullying victim). However, the factor reducing such tendencies in such a situation (e.g., left-behind children) may be social support from the family and/or a good relationship with teachers. This kind of support seems to reduce stress and support young people in solving their problems effectively.

The results of the present study indicate that 96% of children declare help-seeking behaviors, which contrasts with previous findings (29, 33). One of the explanations of that phenomenon may be the specificity of studied group (workshops' participants, volunteers). The results of our research also show that the perception of others, mainly adults, as sources of support is much greater when the adolescent has the experience of a family communicating efficiently, fulfilled, and satisfied with itself. No relationship is a direct cause of trouble. However, it seems that growing up in a family environment conducive to open communication and experiencing satisfaction with being together may foster satisfaction of needs, including the need for attention from others, but also moderate social skills necessary to see potential and real sources of support in other sources. This type of experience turns out to be significantly associated with a lower intensity of violent behavior not only in girls but also in boys (63). A partial reference to the obtained results are reports indicating a relationship between authoritarian forms of upbringing and the occurrence of behavioral difficulties, including the tendency to use bullying (64). According to the findings of Charalompous et al. (64), authoritarian parents favor the acceptance of violent behavior as a form of coping, and in the eyes of children they are not very sensitive to their needs and not very communicative or open to talking about social problems or dangers. Undoubtedly, this gap may be filled by the perception of other adults or peers as sources of support. However, according to the obtained *a posteriori* model of the Bayesian networks, it is more probable with positive experiences in the family system in which one grows up.

According to the obtained model, the probability of noticing and using support in the immediate environment is also higher in adolescent students in a situation of temperamental tendency to associate and stay among people. In this context, it can be concluded that a biologically shaped attitude toward people may favor focusing on relationships with others and perceiving resources in one's environment. This factor may be related to seeking support and help from others, to help them deal with their emotions. As shown by the research by Hunter et al. (34), this type of attitude may be particularly conducive to using assistance when experiencing bullying as a victim. Focusing on “feeling better” may be a strategy and a need (especially in adolescent girls), which should be taken into account when planning aid interventions. Undoubtedly, as the results of the research presented by the authors show, this requires the ability to use the resource, which is the social environment. It can also refer to pro-social features. These, in the opinion of Pouwells et al. (65), may favor being liked in adolescence, despite the lack of a distinctive social position. Moreover, according to the authors, they are more often attributed to youth defending victims or outside youth than to youth acting as victims or perpetrators of bullying.

### Limitations and Future Directions

The research in this article has some limitations. First of all, the research was conducted on a relatively small group, in the age group corresponding to middle adolescence. The youth who participated in the study constituted the group of participants of workshops aimed at preventing the phenomenon of aggression and school violence. The study authors had no direct control over which students attended these workshops and who were excluded from the workshops. The sample was relatively small, but the number of respondents allowed for an initial verification of the possibility of using Bayesian networks for research in the area of bullying and the aspect related to seeking help. In general, Bayesian networks are never fixed, and it can be easily adapted to new observations. When constructing the Bayesian network, the effective sample size depends on how resistant to change the model should be. The higher the assumed resistance should be, the higher the effective sample size should be. The goal of this study was to determine the possibility of using Bayesian networks to predict the behavior of adolescents related to bullying as well as seeking help in a situation of violence. The conducted analyses have shown that even simple Bayesian networks may be used for the correct classification of vast majority of the cases. The networks that have been constructed can be easily adopted in clinical practice, but also verified in future studies.

Self-report tools were also used in the study. The adopted methodology allowed us to learn about the personal experiences and perceptions of adolescents, which seems to be particularly valuable in the situation of looking for predictors of bullying. The obtained results should be treated as a guideline for the use of Bayesian networks in clinical practice. Continuation of research is required to generalize the results to the entire population. In the future, it is worth verifying the obtained results by expanding the research group in terms of gender, age, and behavioral differentiation. The presented research is an interesting proposal for the use of Bayesian networks in screening the diagnosis of victims of persecution and seeking help.

## Conclusions

Bayesian networks were used to analyze the data in this article. The constructed network made it possible to show the influence of variables related to temperament and variables related to the family environment on the probability of bullying or, in fact, a reverse reaction related to seeking help and support. The obtained results and the conducted analyses indicate that the Bayesian network model may be useful in clinical practice.

The network model obtained in the presented study clearly indicates the need to include factors related to the temperament of adolescent children as well as factors related to the relationship and the ability to adapt to the family system in preventive programs. Targeting the strengthening of these aspects, as well as supporting the ability to seek help in the environment seem to be crucial for effective intervention in adolescents using bullying.

## Data Availability Statement

The datasets generated for this study are available on request to the corresponding authors.

## Ethics Statement

The studies involving human participants were reviewed and approved by Ethics Committee of Institute of Applied Psychology, Jagiellonian University in Krakow. Written informed consent to participate in this study was provided by the participants' legal guardian/next of kin.

## Author Contributions

KS-W conceived the study, reviewed and interpreted the results, drafted the manuscript, and provided acquired the funding for publication. KS-W and ZW contributed to the design of the study and organized the database. KS-W and BI organized the data curation. BW performed statistical analysis. KS-W, ZW, and BI administered the project. KS-W, ZW, BW, and BI discussed the results and contributed to the analysis and contributed to the manuscript revision. BI supervised the work and acquired funding for academic translation. All authors have read and agreed to the submitted version of the manuscript.

## Conflict of Interest

The authors declare that the research was conducted in the absence of any commercial or financial relationships that could be construed as a potential conflict of interest.
